# Challenging monogamy in a spider with nontraditional sexual behavior

**DOI:** 10.1038/s41598-022-09777-7

**Published:** 2022-04-08

**Authors:** Fedra Bollatti, Catalina Simian, Alfredo V. Peretti, Anita Aisenberg

**Affiliations:** 1 Facultad de Ciencias Exactas, Físicas y Naturales, Departamento de Diversidad Biológica y Ecología, Universidad Nacional de Córdoba, Córdoba, Argentina; 2 Consejo Nacional de Investigaciones Científicas y Técnicas (CONICET), Laboratorio de Biología Reproductiva y Evolución, Instituto de Diversidad y Ecología Animal (IDEA), Córdoba, Argentina; 3 Departamento de Ecología y Biología Evolutiva, Instituto de Investigaciones Biológicas Clemente Estable, Montevideo, Uruguay

**Keywords:** Evolution, Zoology

## Abstract

Each species and sex can develop different reproductive strategies to optimize their fitness while assigning reproductive effort. *Allocosa* *senex* is a sex-role reversed spider whose males construct long burrows in the sand. They wait for wandering females to approach, assess their sexual partners and donate their constructions to females after copulation. Females stay in the burrow and lay their egg-sac. When offspring are ready for dispersion, females leave the burrow and gain access to new mating opportunities. Males are choosy during mate courtship, preferring to mate with virgin females over copulated ones, which can even be cannibalized if males reject them. This situation turns new mating opportunities dangerous for copulated females. We wondered whether a copulated female inside the previous mate's burrow responds to courtship from a new male and if this new male can copulate, avoiding burrow construction costs. We also explored whether courtship and copulation behaviors during the first sexual encounter affected the probability of occurrence of a second copulation. For that purposes we exposed copulated females inside male burrows to new males (non-donor males). Males could locate and court females inside the previous male's burrow, and females accepted a second copulation. Hence, *A.* *senex* females are not monogamous as was expected but increase their reproductive success by copulating with non-donor males. Also, males can develop opportunistic tactics, suggesting a more dynamic mating system for this sex-role reversed spider than assumed.

## Introduction

Polyandry is a widespread female sexual strategy that increases genetic variability, decreases extinction risk and dilutes the chance of mating failure^1^. It can result in direct benefits for the females such as allowing mate evaluation and choice, receiving nuptial gifts or other resources, increasing fertility, and/or indirect benefits such as ensuring good genes, guaranteeing attractive offspring, among others^1^. However, this female strategy may negatively affect male fitness because it opens the opportunity of female mate choice through biasing paternity in favor of a sexual partner^2,3^.

In consequence, polyandry may induce males to exacerbate direct and/or indirect competitive strategies (e.g., fighting with rivals or performing high mating effort that limits female remating possibilities)^4,5^. Even more, it could lead males to evade rivalry with superior males and develop alternative reproductive tactics (henceforth ARTs)^6,7^. ARTs refer to different ways of obtaining fertilization in both males and females, resulting in selecting certain traits and eliminating intermediate expressions to maximize fitness^6,7^. In this way, some individuals will invest in privileged access to matings through behavioral, physiological, or morphological competition, but individuals who cannot deal with such energetic investment will adopt an alternative mating tactic that increases their own fitness (e.g., satellite males, pseudo-females, coercive matings)^7^.

ARTs have been documented across many taxa such as fishes, amphibians, reptiles, birds, mammals, insects (see taxonomic reviews of alternative reproductive tactics section in Taborsky, 2008)^7^ and arachnids^8,9^. However, the majority of these reports are in species with “conventional” sex roles, constraining the records of males exhibiting ARTs in “sex-role reversed” species only to certain fish^7,10^. This is remarkable given that males generally make a significant reproductive effort in sex-role reversed species, and it might be expected that some males cannot achieve this effort, originating a scenario that would promote the emergence of ARTs.

*Allocosa* *senex* (Mello-Leitão, 1945) is a sand-dwelling wolf spider that exhibits reversal in sex roles and sexual size dimorphism expected for spiders: females are the mobile sex who starts courtship, and males are larger than females^11^. Copulation occurs inside males’ burrows, and after it ends, they donate their own constructions to females as a nuptial gift^11^. It has been indicated that once females copulate with a burrow-donor male, they oviposit there and remain inside until the juveniles are prepared for dispersal^11,12^. Such females accept a second copulation once they exit the burrow for offspring dispersal, thus establishing a scenario of temporary monogamy^14^. Moreover, copulated females will need to copulate again to obtain a new burrow for the next oviposition, since they can lay up to four consecutive egg-sacs after one copulation, but they are not good diggers^13,14^. However, this is a scenario where *A.* *senex* males often cannibalize females, since they prefer to copulate with virgin females in good body condition^15^. Regarding males, burrows are essential to them not just because they are shelters during daylight and winter, but also because burrows are the target of female choice^11^. Burrow construction negatively affects males’ weight^16^, making their donation costly in terms of energetic demands. Also, *A.* *senex* habitat is seasonal, harsh and unstable (marine and river coasts of South America^17^); consequently, this creates the ideal evolutionary conditions for the emergence of ARTs to evade male burrow construction costs.

Here we tested, under laboratory conditions, female sexual receptivity to copulation with a second male in a prior male’s burrow and male’s ability to locate and court them. Thus, due to the general benefits of polyandry^1^ and for avoiding attacks by large donor males^15^, it could be expected that copulated females inside previous males’ burrows would respond to non-donor males courtship and copulate with them. We also expect that at least some males that cannot cope with the burrow building would be able to locate and court females inside previous males’ burrows, as a way to avoid construction costs and to maximize their fitness. In addition, we explored the effects of several behavioral variables and individual traits related with the first sexual encounter (courtship and copulation durations, number of mounts, female body shakes, and ejaculations, female and male body size, and female age) on courtship and copulation success during a second sexual encounter. Generally, females are more receptive when virgin, but become choosier after mating^18,19^. This increased selectivity in female spiders has been related to her own size^20^ and age^21^, the size of the male^22^, copulatory courtship behaviors^23^, the duration of copulation^24^ and sperm transfer^25^. Specifically, the peculiar existence of repeated mountings during the copulation in *A.* *senex* could indicate stimulation and copulatory courtship to females^11^. Regarding females, the body shakes performed during copulation have been reported as a communication signal to motivate more frequent ejaculations by the male^26^. We expect that first sexual encounters with longer durations, higher number of mounts, female body shakes and ejaculations generate more female sexual reluctance to copulate with a second male (i.e. decreasing the probability of a second copulation). On the other hand, older females will be more receptive to re-copulation as a way to capitalize the few opportunities left. Finally, larger body sizes in females and males could be related to greater fecundity in females, and higher competitive characteristics in males, affecting female re-copulation responses.

## Results

### Courtship and copulation probabilities in first and second female sexual encounters

**Figure 1:**
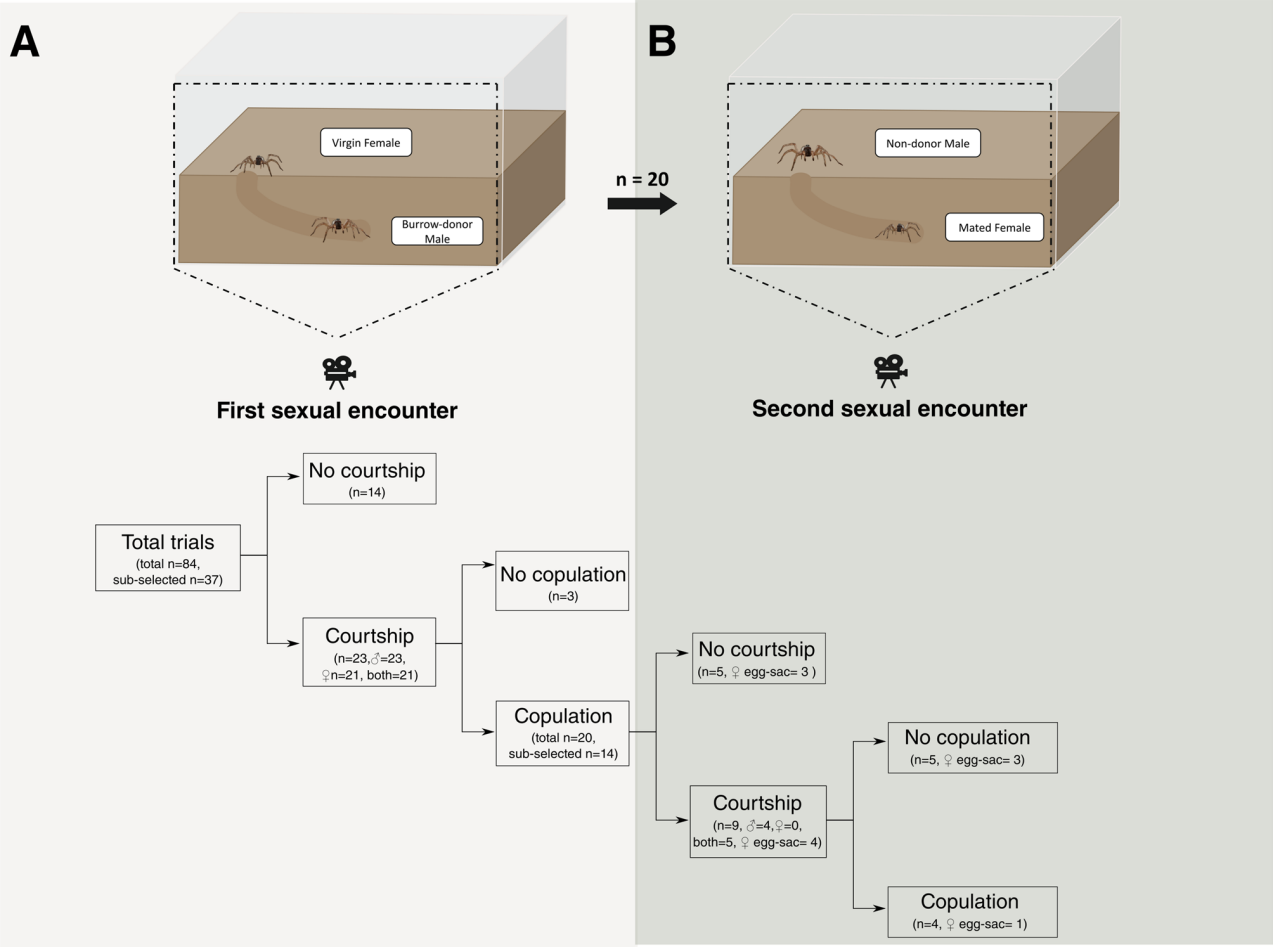
Scheme of the experiment (above) and flow chart of trials outcome (below). The illustration on (**A**) represents the first sexual encounter with a burrow-donor male, and the illustration on (**B**) represents the second sexual encounter with a non-donor burrow male. Arrows indicate the progression of events and/or the circumstances in which they occurred. The numbers of trials are given in parentheses. Since in numerous trials some individuals did not interact with each other and/or did not perform any movement at all, to calculate the real probabilities of courtship and copulation during the sexual encounters, we sub-selected from the total number of interactions those in which at least one of the individuals noticed the other one. We considered the following indicator criteria: (1) the individual remained outside the burrow entrance for at least 5 s, and/or (2) the individual inside the burrow moved towards the burrow entrance.

Of the total interactions selected for analysis (n = 37) approximately 60% involved courtship of either sex, and 50% resulted in copulation. Of this total of initial interactions, between 13 and 25% courted in a second sexual encounter and only 10% had a second copulation. Of the total number of sub-selected first copulation interactions (n = 14) between 35 and 65% courted, and 30% reached a second copulation (Table 1, Fig. 1).

**Table 1:** Courtship and copulation proportions during first and second sexual encounters, with the corresponding results of the statistical comparisons between the two groups.

	First sexual encounter	Second sexual encounter	Comparison
Female courtship	0.57 (n = 21 from 37)	0.36 (n = 5 from 14)	χ^2^ = 2.412; p = 0.120
Male courtship	0.62 (n = 23 from 37)	0.64 (n = 9 from 14)	χ^2^ = 0.023; p = 0.880
Copulation	0.54 (n = 20 from 37)	0.29 (n = 4 from 14)	χ^2^ = 2.367; p = 0.124

**Figure 2:**
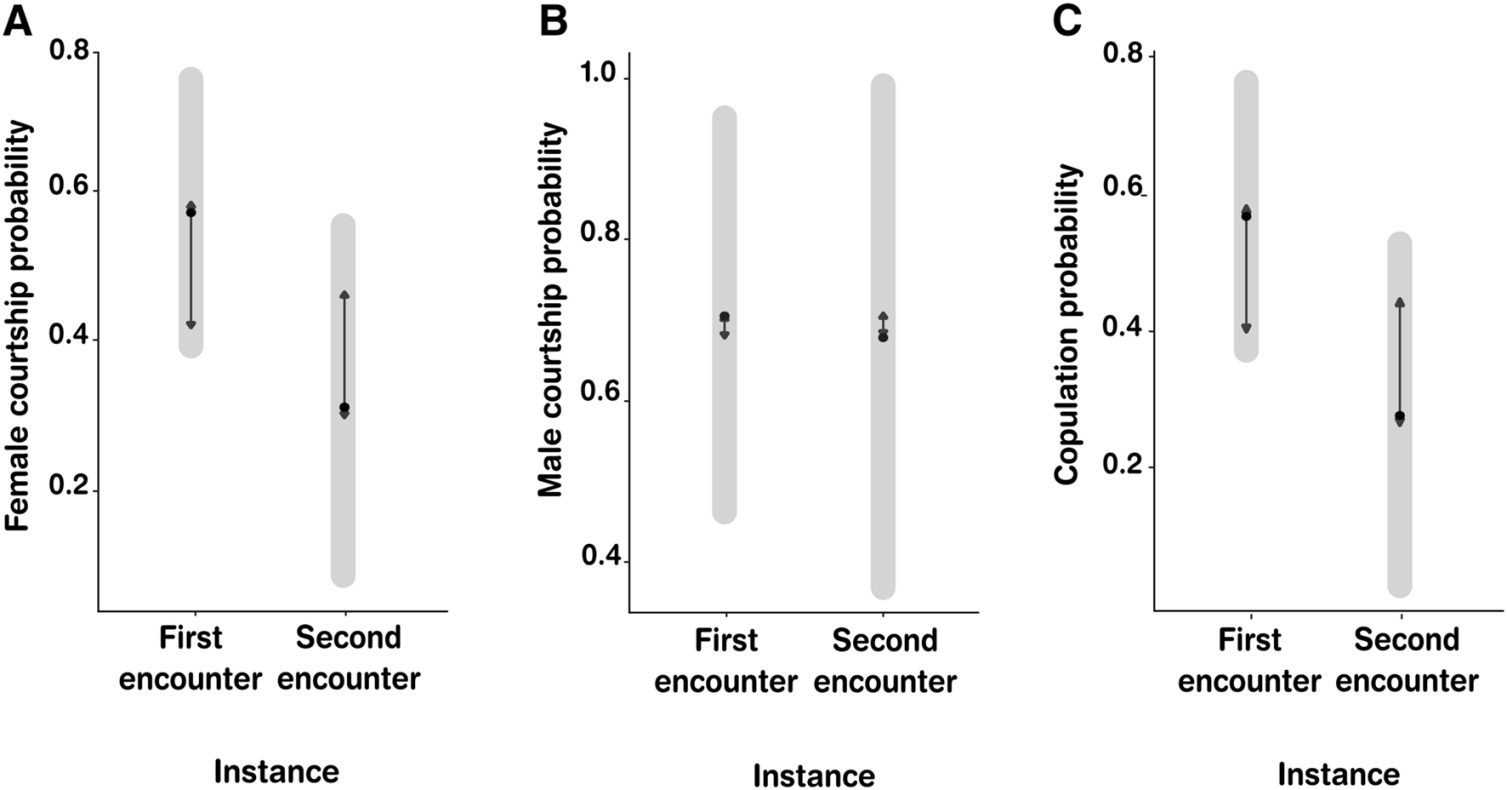
Courtship and copulation probabilities during first and second sexual encounters of behavioral trials. (**A**) Female courtship probability, (**B**) male courtship probability, (**C**) copulation probability. The gray bars represent the confidence intervals of the marginal mean and black arrows stand for comparisons between means. If the arrow of one mean overlaps with another one, it means there were no significant differences according to Tukey's test and with an alpha value of 0.05.

*Allocosa* *senex* females courted in similar proportions during first sexual encounters and second sexual encounters, as did males (Table 1, Figs. 1 and 2A,B, see corresponding courtship ethograms at Supplementary Fig. 1). We also found that female copulation probability was non-significantly different between first and second sexual encounters (Table 1, Figs. 1, and 2C, see corresponding copulation ethograms at Supplementary Fig. 1).

### Copulation behaviors in first and second female sexual encounters

We detected differences in the sequence of behaviors during copulation (taken from Ref.^26^) in first and second female sexual encounters (Supplementary Fig. 1) and recorded the occurrence of new sexual behaviors during second sexual encounters.

During courtship in the first sexual encounter it was not frequent that males pulled their legs outwards to touch the female before they entered to the burrows^26^, while during the second sexual encounter most of the females pulled their legs outside the burrow in order to touch the visiting male (Supplementary Video 1). We observed male–female swicht positions behavior (male and female exchange positions inside the burrow, while the male moves to the top, the female moves to the base of the burrow^26^) in all copulations during the first sexual encounter; however, this behavior did not occur during second sexual encounters (see ethograms at Supplementary Fig. 1, Supplementary Video 1). Also, we did not record any case of cannibalism of copulated females during second sexual encounters. Males never entered inside the burrow if females did not perform any courtship previously (Supplementary Video 2).

We summarize and compare numbers and durations (mean ± SD) of sexual behaviors performed during first and second sexual encounters in the Supplementary Table 1.

### Analyses of factors shaping courtship and copulation in second sexual encounters

When testing the effects of life history traits on the probability of courtship in the second sexual encounter, the best-fitted model was the null, so none of the variables was predictive of our response variable (Supplementary Table 2A). In the case of sexual behavior variables, the best model included two behaviors: number of female shakes and number of mounts of the first sexual encounter. The number of female shakes was negatively related to the probability of male courtship in the second sexual encounter (χ^2^ = 4.084; p = 0.043), but the number of mounts had only a marginally significant influence (χ^2^ = 3.383; p = 0.066). Copulation probability in the second sexual encounter was not explained by any life history variable, with the null model having the lowest AIC (Supplementary Table 2B). Finally, when analyzing the effect of behavioral variables, we found two equally fitted models: the null model and the model with the duration of copulation at the first encounter. However, this variable did not appear to be statistically significant (χ^2^ = 2.589; p = 0.108).

## Discussion

According to the results of the current study *A.* *senex* females respond similarly to courtship when facing a male who offers his own refuge as nuptial gift compared to a male who visits her at the burrow constructed by her previous mating partner. Furthermore, we did not find significant differences between the probability of copulation and re-copulation. This pattern indicates that although females are choosy as virgins, they are capable of recopulating before egg-sac construction and even with the egg-sac hanging from her spinnerets; this differs from what had previously been reported for this species^11,12^. We also found that males may display different reproductive tactics to avoid the costs of donating their burrows and maximize their mating opportunities.

Although there are numerous examples of females that copulate with more than one male across the animal kingdom^27^, including spiders^28^, the outstanding characteristic of *A.* *senex* females is that they can copulate more than once despite the high male reproductive investment. Such findings suggest a conflict of interest between sexes in this species, in which male attempts to ensure paternity faces female polyandric strategy^29^. Under another perspective, considering that *A.* *senex* is a spider in which males are larger than females and they can cannibalize them when they are not virgins^15^, the present results could be framed in a scenario of polyandry for convenience^1,30–32^, similar to what has been described for the dung fly *Sepsis* *cynipsea*^33^, the African butterfly *Bicyclus* *anynana*^34^, the water strider *Gerris* *buenoi*^35^, and the desert spider *Stegodyphus* *lineatus*^36^, among others. In this case, females would re-copulate to avoid the cost of resisting, which could be more expensive than the cost of mating^1,14,15^. Furthermore, females of second sexual encounters were located inside the burrows of their previous sexual partners, and resisting would potentially place their lives at risk^1^. However, no cases of male cannibalism were detected during the development of this study. Also, no male entered burrows without prior female courtship, which means that the mere fact that one searching male is present does not determine that another copulation will occur. Previous authors^11^ discussed that *A.* *senex* male behavior of closing the burrow could function to avoid attacks from other males on the female that remains in the donated burrow. However, the risk of male sexual cannibalism seems to be low under these circumstances according to the current study. Another possible explanation is that females are choosing different traits according to distinctive scenarios.

On the other hand, the fact that females can copulate in a second sexual encounter provides the basis for post-copulatory sexual selection processes to occur^2,3,5^; however, the intensities could be different depending on the presence/absence of the egg-sac; a female with an egg-sac could be receptive to re-copulate since she has already used part or all sperm of the previous mate, and accepting a new male inside the burrow could imply compromising her survival^14,15^. Otherwise, a female without an egg-sac may be receptive to copulate again because of the known benefits of polyandry (i.e. direct benefits—nuptial gifts, increased fertility, resources, etc.—and indirect benefits—good genes, attractive offspring, etc.)^1^.

Our results also showed that *A.* *senex* males can locate, court, and copulate with females located within the burrows of their previous sexual partners. They courted females regardless of their reproductive condition and context (visiting female or inside another male’s burrow), revealing a more dynamic mating system than was previously assumed^11,14^. Males could opt for constructing energetically demanding structures for mate attraction and as paternal effort^37^—burrow-donor males—or omit these costs and exploit their competitors' investment to gain access to new mates—non-donor males—^38–40^. In the different scenarios, male energetic investment would possibly not be the same nor its reproductive outcomes. For example, the tactic of building and donating burrows would be energetically demanding, but at the same time, the male would ensure higher paternity chances by mating with virgin females in good body condition. *A.* *senex* belongs to the group of entelegyne spiders for which sperm priority has been reported to be biased towards the first males^41,42^; however, see Refs.^43–45^. In addition, there are reports from this species indicating that the first egg-sac of the female is the most successful in terms of the number of eggs^46^. In contrast, non-donor males would invest less energy in construction since they do not donate their burrow and instead exploit the burrow of a donor male, but they invest in mate searching, thus ensuring a percentage of paternity.

Female shakes during copulation in first sexual encounters affected negatively the probability of male courtship during a second sexual encounter. According to previous studies^26^, female body shakes would be related with higher sperm transfer, so, these females would be less receptive to re-copulate in a second encounter. Helsdingen^47^ proposed that sperm and/or associated substances can alter female receptivity in spiders, which was confirmed years later in another wolf spider—*Schizocosa* *malitiosa*^25^. Therefore, those females would not emitting sexual pheromones that trigger male courtship. However, in this study we observed cases in which the male courted and the female did not respond, so more studies are needed to make accurate interpretations. On the other hand, numerous life history and sexual behavior variables that we predicted would have an effect on the probability of courtship and copulation of second sexual encounters did not have effects or were not selected as weight variables in the final models. This could be suggesting that there are other variables that are affecting these events which we have not considered in this investigation.

According to Oliveira et al.^7^, ARTs can be fixed or dynamic, and this is related to stable or unstable, both physically and socially, mating environments. Dynamics ARTs are related to plastic responses to conditions and have been reported in fish^39^, anurans^48^, and birds^49^. In the wolf spider *Rabidosa* *punctulata*^50^ males can alter their mating tactic expression depending on female mating status. They court virgin females and mount without courtship already copulated females, a tactic that larger males with better body condition more frequently perform. Another example has been documented in the spider *Pisaura* *mirabilis*, in which males can perform three different mating tactics: offering a prey gift, offering a non-nutritious gift, or not offering any gift depending on prey availability and sperm competition intensity^51^. Scott et al.^52^ reported that in *Latrodectus* *hesperus* males can perform conventional tactics for mate search, courtship and copulation, or they can adopt mate guarding and mating with immatures as the reproductive season progresses. Although the results here obtained do not allow us to be certain in which of these alternative reproductive tactics best fit our findings, it is important to note that *A.* *senex* has been reported in environments that vary in terms of environmental conditions and factors such as density or operative sexual proportion^53^; however, further studies are needed to answer this question.

The present results make this species even more interesting than before. We confirm that females of *A.* *senex* can copulate more than once before building the egg-sac and even after egg-sac construction. This would settle precedents of a potential scenario of sperm competition and cryptic female choice. Also, female choice and sexual receptivity would be critical when copulation opportunities arise, and probably these and other factors shape the arisal and maintenance of the described male reproductive tactics. This is consistent with Alonzo and Wagner^54^ hypothetical modelling, which postulated that female choice might alter or delete the expression of male ARTs. Finally, to our knowledge, this could be the first report not only in invertebrates, but also in terrestrial animals with sex-role reversal where alternative reproductive tactics in males are also reported. Future studies will test if these are really male alternative reproductive tactics, and if so, are they fixed or dynamic? Also, it will confirm if there is an effect on the paternity of males adopting one or the other strategy, and, consequently, if the females perform any type of post-copulatory female choice.

## Methods

### Spider collection

We performed eight field trips from October to March 2016–2018 in the localities of La Bolsa (31° 43′ 23.9″ S, 64° 25′ 24.5″ W), Copina (31° 33′ 0″ S, 64° 42′ 0″ O) and Nono (31° 47′ 51.7″ S, 64° 59′ 17.5″ W), Córdoba Province, Argentina. We collected 172 sub-adults and 38 adult males individuals of *A.* *senex* by nocturnal manual sampling^55^. The individuals were located using headlamps while walking or leaning out from the burrow entrances. As *A.* *senex* has been categorized as priority species for conservation^56^, we used moderate samples sizes to avoid extracting too many specimens from nature.

### Maintenance in laboratory

Individuals were maintained at the Laboratorio de Biología Reproductiva y Evolución of IDEA (UNC-CONICET), Argentina. Each individual was conditioned in 5 × 9 × 2 cm boxes, with sand from the original location as substrate and a wet cotton to provide water and moisture. Individuals were fed twice a week with larvae of *Tenebrio* sp. (Coleoptera, Tenebrionidae) and daily monitored to record molt occurence in order to determine the exact time at which individuals reached sexual maturity. After the experiments, voucher specimens were deposited at the Laboratorio de Biología Reproductiva y Evolución, LABRE-Ar, Instituto de Diversidad y Ecología Animal, Facultad de Ciencias Exactas, Físicas y Naturales, Universidad Nacional de Córdoba, Argentina.

### Copulation trials

For behavioral experiments, we used 15 × 30 × 40 cm transparent glass terraria, with a wet cotton to provide water and moisture, and sand from the original location as substrate (a layer 15 cm deep of dry sand on the surface, and a layer 5 cm deep of moistened sand at the base)^57^. We used virgin females 7–20 days of adult age, coinciding with the period of sexual receptivity reported for this species^58^. Virgin males were used seven days after reaching adulthood in the laboratory and males with unknown reproductive history (captured as adults) were used 7 days after being collected.

#### First sexual encounter

We randomly assigned females and males to experimental pairs. Males were placed in the terrarium 48 h before the trials to allow burrow construction. Generally, males of this species dig against glass walls, allowing observation still inside their burrows^11^. We used for the first sexual encounters only those males which constructed burrows in the period defined. The trial began when each female was introduced to the terrarium. If after 30 min individuals did not engage in courtship, we ended the trial. If courtship occurred in that period by either—male or female—but in the next 30 min (i.e. 1 h after the beginning of the trial) individuals did not engage in copulation, we ended the trial. Finally, if copulation occurred, we ended the trial after the male left the burrow. Then, each trial had one of these four possible outcomes: courtship (by male and/or female), no courtship, copulation and no copulation. Virgin males that did not court were reused with up to two more virgin females (Fig. 1A).

#### Second sexual encounter

We allowed the already copulated females n = 20 (7 carrying egg-sacs and 13 not carrying egg-sacs) to remain 1–6 days inside the donor-male burrow, and then a new randomly assigned male with unknown reproductive history (field males) was introduced to the terrarium. According to previous studies, males with unknown reproductive history (captured as adults) or virgin males (molted under laboratory conditions) do not show differences in courtship or copulation behavior in this species^15^. The trial began when each male was introduced to the terrarium. If after 30 min individuals did not engage in courtship, we ended the trial. If courtship occurred in that period by either—male or female—but in the next 30 min (i.e. 1 h after the beginning of the trial) individuals did not engage in copulation we ended the trial. Finally, if copulation occurred, we ended the trial after the male left the burrow. Then, each trial had one of these four possible outcomes: courtship (by male and/or female), no courtship, copulation and no copulation (Fig. 1B). In this sexual encounter we considered male courtship (and not female courtship), since the number of courting males was higher and the number of courting females was equal to the number of copulations.

We conducted all the trials in total darkness, recording the sexual interactions with Sony DCR-SR85 and Sony Handycam DCR-SR65E digital videocameras, both equipped with night-shot function. We analyzed the recordings with JWatcher software^59^.

Regarding sexual behavior traits, we recorded courtship and copulation durations, number of mounts, female body shakes, and number of ejaculations during first and second sexual encounters, following^11,26^ (see Fig. 1). Female body shakes have been reported as signals that can elicit palpal insertions and ejaculations in this species^26^. The number of ejaculations was indirectly recorded as erections of male leg spines which are related to hematodocha inflations, following previous studies on this species^11^.

Since in numerous trials some individuals did not interact with each other and/or did not perform any movement at all, to calculate the real probabilities of courtship and copulation during the sexual encounters, we sub-selected from the total number of interactions those in which at least one of the individuals noticed the other. We considered the following indicator criteria: (1) the individual remained outside above the burrow entrance for at least 5 s, and/or (2) the individual inside the burrow moved towards the burrow entrance.

Regarding life history trait variables, we measured prosoma width—an indicator of body size in spiders^60,61^—and weight of all individuals to calculate body condition indices (BCI = residuals of a linear regression between prosoma width and weight)^62^. The weight of the individuals was measured at the end of the experiments. We also measured female age (calculated as the number of days as an adult from the last molt until the trial), and we considered the occurrence of egg-sac for the second sexual encounter.

### Statistical analyses

Data were analyzed using generalized linear mixed models (GLMM) in R 3.6.1 statistical software^63^. In all the cases, the distributions of response variables were explored graphically using the Fitdistrplus package^64^ and analytically using the Akaike information criterion. Before modeling, we corroborated collinearities of explanatory variables using the “cor.test” function to avoid overlapping effects. We started with models containing the additive effects of all the predictive variables which were subjected to variable selection using the MuMin package^65^, and employing the Akaike information criterion corrected for small samples (AICc). For model building, we used the function “glmer” from lme4 package^66^. For courtship probability during second sexual encounters we added the female and both males BCI, female age and the presence of egg-sac (life history traits variables) in one model, and in a second model we added courtship and copulation durations, number of mounts, female body shakes (sexual behavior traits). For copulation probability during second sexual encounters we added the female and both males BCI, female age and the presence of egg-sac (life history traits variables) in one model, and in a second model we considered courtship and copulation durations and number of ejaculations (sexual behavior traits). We only considered the variables selected by this process in the elaboration of the final models (see Supplementary Table 2).

#### Courtship and copulation probabilities in first and second female sexual encounters

In independent models, we set as response variables the male courtship, the female courtship and the copulation probabilities on each encounter, which were binomially coded (1: occurred, 2: did not occur). The instance of sexual encounter (first sexual encounter, second sexual encounter) was added as a fixed effect to assess whether there were differences in the probability of occurrence of these events between both sexual encounters. We included female identity (because each experienced two sexual encounters) and male identity (due to the re-use of some males in the first instance) as random effects. We used emmeans package^67^ to compare the estimated marginal means of response variables among first and second sexual encounters for the graphics showed in Fig. 2.

#### Factors shaping courtship and copulation in second sexual encounters

Similarly to 4.4.1, we considered in independent models binomially the courtship and copulation probabilities (1: occurred, 2: did not occur), but only in the second sexual encounter. The variables selected for the models to analyze the determinants of courtship on second sexual encounters were the number of mounts and the number of female body shakes during first sexual encounters. Instead, the variables selected for the copulation probability models in second sexual encounters were: the number of ejaculations and the copulation duration during first sexual encounter. The latency between copulation among first and second sexual encounters was considered as a random effect (1 to 6 days) in both models.

### Ethics declarations

All the field animal collections were performed in Argentina. Despite no certification or approval is needed for studies involving invertebrates in Argentina, we collected the minimal sample size necessary for the descriptions explained above and kept them in the laboratory under similar natural conditions. The research was based on non invasive behavioral observations.

## Supplementary Information

Supplementary Information.

Supplementary Video 1.

Supplementary Video 2.
