# Supplementary material for: Challenging monogamy in a spider with nontraditional sexual behavior
Source: Sci Rep. 2022 Apr 8;12:5948. doi: 10.1038/s41598-022-09777-7 (PMC8993839; doi:10.1038/s41598-022-09777-7)
Supplement: Supplementary file 1 — Supplementary Information. [file 41598_2022_9777_MOESM1_ESM.docx]

## Supplementary Figure 1. Schematic of *Allocosa senex* copulation, (A) during first sexual encounter (modified from Garcia Diaz et al. 2015) and (B) during second sexual encounter (this study). The table with probabilities is common to both ethograms.

## Supplementary Figure 1.

##
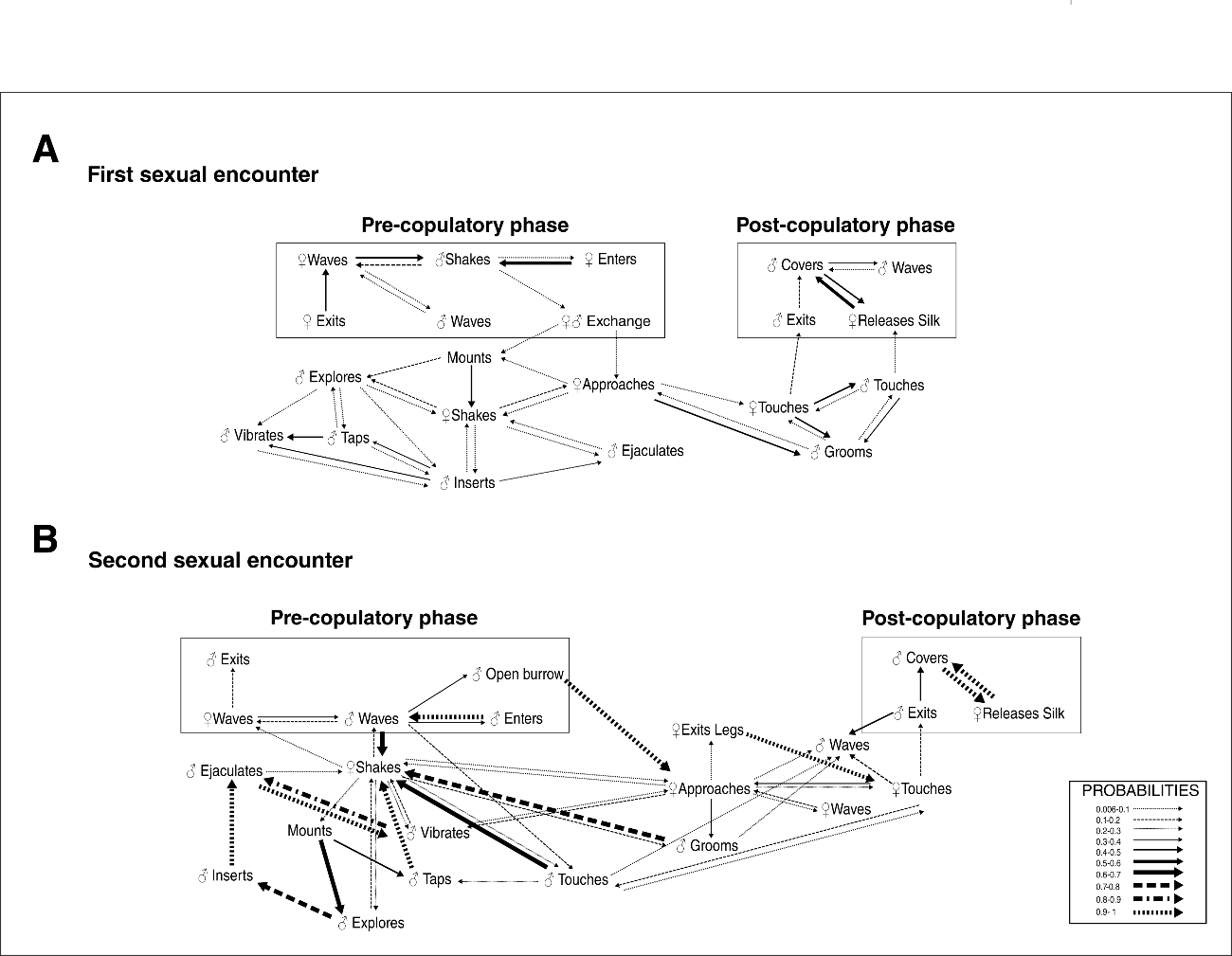


**Supplementary Table 1.** Data (mean ± SD) of the number of mounts, palpal insertions, female body shakes, number of ejaculations, and courtship and copulation durations (sec) during first and second sexual encounters.

|  | **First sexual encounter** | **Second sexual encounter** |
| --- | --- | --- |
| **Number of Mounts** | 7.67 ± 2.54 (n = 17) | 5 ± 2. 56 (n = 3) |
| **Number of palpal insertions** | 11.72 ± 6.08 (n = 17) | 7 ± 3.46 (n = 3) |
| **Number of female shakes** | 147.12 ± 104.66 (n = 17) | 158 ± 52.83 (n = 3) |
| **Number of ejaculations** | 160.44 ± 69.37 (n = 15) | 204 ± 61.58 (n = 3) |
| **Courtship duration** | 565.12 ± 709.63 (n = 20) | 684.33 ± 847.45 (n = 4) |
| **Copulation duration** | 1956.39 ± 496 (n = 20) | 1757.55 ± 518.20 (n = 4) |

**Supplementary Table 2.** Top 5 candidate models after model dredging to test A) the effect of individuals life history and sexual behavior traits on male courtship probability during second sexual encounters, B) the effect of individuals life history and sexual behavior traits on copulation probability during second sexual encounters. We show AICc and log-likelihood value (logLik), freedom degree (df), AICc weights for models and differences in AICc values (ΔAICc). Bold values indicate the best-fitted models (ΔAICc<2)**.** Abbreviations: '+': additive effects only; '1| ': random effects; fse: first sexual encounter.

| **Response variable** |  | **Model structure** | **ΔAICc** | **AICc** | **weight** | **df** | **logLik** |
| --- | --- | --- | --- | --- | --- | --- | --- |
| **A**. **Male courtship probability during second sexual encounters** |  | 1 + (1\| latency) | **0.0** | **19.6** | **0.492** | **2** | **-6.931** |
|  |  | BCI second male + (1\| latency) | 3.13 | 22.7 | 0.103 | 3 | -6.355 |
|  | **Life history traits** | BCI female + (1\| latency) | 3.39 | 23.0 | 0.090 | 3 | -6.482 |
|  |  | Female age + (1\| latency) | 3.47 | 23.0 | 0.087 | 3 | -6.521 |
|  |  | BCI first male + (1\| latency) | 3.52 | 23.1 | 0.085 | 3 | -6.549 |
|  |  | Shakes number + mounts number + (1\| latency) | **0.00** | **26.5** | **0.389** | **4** | **-7.736** |
|  |  | Shakes number + (1\| latency) | 2.07 | 28.6 | 0.138 | 3 | -10.450 |
|  |  | 1 + (1\| latency) | 3.20 | 29.8 | 0.078 | 2 | -12.477 |
|  | **Sexual behavior traits** | Shakes number + mounts number + fse copulation duration + (1\| latency) | 3.47 | 30.0 | 0.069 | 5 | -7.510 |
|  |  | Shakes number + mounts number + fse courtship duration + (1\| latency) | 3.62 | 30.2 | 0.064 | 5 | -7.583 |
| **B.** **Copulation probability during second sexual encounters** |  | 1 + (1\| latency) | **0.0** | **15.7** | **0.529** | **2** | **-5.004** |
|  |  | BCI female + (1\| latency) | 3.64 | 19.4 | 0.086 | 3 | -4.680 |
|  | **Life history traits** | Female age + (1\| latency) | 3.64 | 19.4 | 0.086 | 3 | -4.680 |
|  |  | BCI first male + (1\| latency) | 3.72 | 19.4 | 0.082 | 3 | -4.720 |
|  |  | Egg-sac+ (1\| latency) | 3.84 | 19.6 | 0.079 | 3 | -4.780 |
|  |  | 1 + (1\| latency) | **0.00** | **20.4** | **0.311** | **2** | **-7.721** |
|  |  | Fse copulation duration + (1\| latency) | **0.02** | **20.4** | **0.309** | **3** | **-6.190** |
|  | **Sexual behavior traits** | Fse copulation duration + fse courtship duration + (1\| latency) | 2.05 | 22.4 | 0.112 | 4 | -5.388 |
|  |  | Fse courtship duration + (1\| latency) | 2.35 | 22.7 | 0.096 | 3 | -7.356 |
|  |  | Ejaculations number + (1\| latency) | 2.99 | 23.4 | 0.070 | 3 | -7.678 |

## Supplementary video 1. *Allocosa senex* copulation among a virgin female and burrow-donor male, and re-copulation among a already copulated female with a non-donor male.

## Supplementary video 2. Videos showing the active female participation on the re-copulation occurrence. In the first video, we observe a non-donor male courting at the burrow entrance and the female not responding to its behavior. In the second video, we observe a non-donor male courting and a female rejecting and chasing him. In the third video, we observe a mated female courting a non-donor male from inside the burrow and the male not responding to its behavior.
